# Evaluation of Haematological Parameters and Uric Acid in the Diagnosis of Late Onset Neonatal Sepsis

**DOI:** 10.7759/cureus.39691

**Published:** 2023-05-30

**Authors:** Zeynep Yılmaz Oztorun

**Affiliations:** 1 Department of Pediatrics, Omer Halisdemir University School of Medicine, Nigde, TUR

**Keywords:** mean platelet volume, eosinophil, uric acid, neutrophil/lymphocyte ratio, late neonatal sepsis

## Abstract

Introduction

A number of parameters studied in a whole blood count can be helpful in the diagnosis of neonatal sepsis. The platelet/lymphocyte ratio (PLR) is a systemic inflammatory marker in early sepsis and has been used as a diagnostic indicator in cardiovascular events and cancer. Being one of the major antioxidants in human biological fluids, serum uric acid is responsible for neutralising free radicals. The red cell distribution width/platelet ratio (RPR) is a diagnostic marker in adult inflammatory diseases. The objective of our study is to investigate the relationship of late neonatal sepsis with whole blood count parameters and serum uric acid levels.

Materials and methods

Newborns older than postnatal three days who had clinical and laboratory findings of sepsis were included in the study. The study included 140 newborns who were divided into three groups, 53 in the culture-proven late sepsis group, 47 in the clinical sepsis group, and 40 in the healthy control group. The whole blood count parameters and serum uric acid levels were examined in both the clinical sepsis and proven sepsis patients at the time when they were diagnosed with sepsis.

Results

The birth week was significantly lower in the evidenced and clinical sepsis patients compared to the healthy control group. Development of late sepsis was significantly higher in the male gender than in healthy controls. Serum uric acid levels were significantly higher in proven or clinical sepsis than in healthy controls. The level of serum uric acid (3.77±1.6) in proven sepsis was significantly higher than the control group (2.83±1.1). The uric acid level had an area under the curve (AUC) 0.552-0.717, 35% sensitivity, 95% specificity, 94.6% positive predictive value (PPV), and 36.9% negative predictive value (NPV) in the diagnosis of proven and clinical late sepsis. Neutrophil/lymphocyte ratio (NLR) was significantly higher in proven sepsis than in healthy newborns and was higher in the clinical sepsis group than in the proven sepsis group (p: 0.002). While the mean eosinophil value was 618.5±472.1 in proven sepsis, it was 549.3±294.9 in the control group and there was a statistically significant difference between the two groups (p: 0.036).

Conclusion

In late-onset neonatal sepsis, the NLR level was higher, and the eosinophil level was lower in the clinical sepsis patients than in healthy newborns. We believe that a higher level of serum uric acid in sepsis is effective in the early diagnosis of patients who also had other clinical findings of sepsis.

## Introduction

Sepsis in the neonatal period is agreed to be a systemic inflammatory response syndrome developing as a result of an evidenced or suspected infection [1]. It is still considered a major problem in newborn intensive care units and leads to significant morbidities and mortalities [2]. Up to 10% of infants experience infection in the first month of their lives; this condition causes 30%-50% of newborn deaths in developing countries [3].

Neonatal sepsis is categorised based on blood culture being positive or not, and the time of its onset. It is agreed to be the proven sepsis in newborns whose blood culture is positive and who have the clinical and laboratory findings of an infection/inflammation; to be clinical sepsis if the blood culture is negative but there is an increase in clinical and inflammatory findings and biomarkers [4]. Neonatal sepsis is divided into two based on the starting time of its symptoms as early-onset sepsis and late-onset sepsis. While early neonatal sepsis starts within the first 72 hours of life, sepsis seen between 72 hours and 28 days is called late neonatal sepsis. Blood culture is agreed to be the gold standard in its diagnosis. The finalization of a blood culture being slow and the presence of false negative results are its limiting sides [5,6]. In the literature, C-reactive protein (CRP), mean platelet volume (MPV), and neutrophil/lymphocyte ratio (NLR) has been used as parameters for helping the diagnosis of neonatal sepsis. The platelet/lymphocyte ratio (PLR) is reported to be a useful systemic inflammatory marker in early sepsis and a diagnostic indicator in cardiovascular events and cancer [7]. Serum uric acid is one of the major antioxidants in human biological fluids and is responsible for the neutralization of more than 50% of the free radicals in the blood [8]. Early and late sepsis differentiation has not been made for uric acid. In the presence of inflammation, proinflammatory cytokines affect the life process of erythrocytes, allowing new and larger reticulocytes to enter the circulation and increasing the red cell distribution width (RDW) [9]. The red cell distribution width/platelet ratio (RPR) is a major systemic inflammatory marker and is helpful in the diagnosis of inflammatory diseases [10]. The objective of our study is to investigate the relationship of late neonatal sepsis with whole blood count parameters and serum uric acid levels.

## Materials and methods

The data of this retrospective case-control study were collected from the computer records of the patients hospitalised for late sepsis in the tertiary Newborn Intensive Care Unit of Niğde Ömer Halisdemir University Training and Research Hospital between April 2017 and April 2020. Newborns who had a major congenital anomaly, congenital infection, early-onset sepsis, and intrauterine growth retardation were excluded from the study. Newborns older than postnatal three days who had laboratory findings of clinical sepsis and sepsis were included in the study. The study included 140 newborns who were divided into three groups, 53 in the culture-proven late sepsis group, 47 in the clinical sepsis group and 40 in the healthy control group. The inclusion criteria for the healthy control group were those whose acute phase reactants (CRP) were tested and negative, whose blood cultures were negative, and whose had no clinical sepsis symptoms.

At least two clinical and two laboratory criteria were used as the basis for a sepsis diagnosis. Screening for sepsis involves obtaining a complete blood count (CBC), CRP, uric acid level, and blood culture results. Since our center has a high rural population density, hygienic conditions were not good and child follow-up visits were not enough, lab investigations are performed routinely in healthy controls. Laboratory investigations were performed to rule out infective pathologies in most of the healthy newborns who presented with complaints of irritability, clamoring to cry and who did not have the opportunity to measure temperature at home. The clinical criteria included hypothermia or hyperthermia, tachycardia (heart rate >160/min) or bradycardia (heart rate <60/min), increased need for oxygen, recurrent apnoea, feeding intolerance, lethargy, and hypotonia; the laboratory criteria included white blood cell count <5x10^9^ cells/L or 20x10^9^ cells/L, immature/total neutrophil count >0.2, platelet count <100x10^9^/L, and CRP >10 mg/L. Patients with a late diagnosis of sepsis from hospital records were screened. From these data, those with appropriate clinical and laboratory criteria meeting the diagnosis of sepsis were selected. Patients who met only the clinical and laboratory criteria were considered to have clinical sepsis and those who, alongside meeting the clinical and laboratory criteria, also had a positive blood culture were considered to have culture-proven sepsis.

The Statistical Package for the Social Sciences (SPSS) 25.0 package program was used for the statistical analysis of the data. Categorical measurements were summarized as numbers and percentages and continuous measurements as means and standard deviations (as medians and minimums-maximums when necessary). The chi-square test was used for comparisons of categorical propositions. The parameters studied were tested for normal distribution using the Kolmogorov-Smirnov test. One-way ANOVA was used for the parameters showing a normal distribution and Kruskal Wallis for those not showing a normal distribution. Based on the clinical sepsis and proven sepsis groups of the patients, the sensitivity and specificity values of RDW/platelet, platelet/lymphocyte, and uric acid values were calculated, and a cut-off value was found based on the area under the ROC curve. Spearman’s rho correlation test was used to find the relationship between the continuous measurement parameters. Statistical significance was set at 0.05 in all tests.

## Results

Of the 140 newborns included in the study, 81 were boys and 59 girls. Due to the retrospective nature of our study, the gender distribution was not homogeneous. Larger series of studies are needed to evaluate the effect on the outcome. The number of patients who were born through spontaneous vaginal delivery was 63 and that of the patients delivered with caesarean section (c/s) was 77. The birth weeks of the patients were found to be 38.5±0.2 weeks, birth weights 3171.2±359 grams, and postnatal days 6.63±3.6. The birth week was significantly lower in patients with proven sepsis and clinical sepsis than in healthy controls (p: 0.001). There was decreased sucking in 79.2% of the patients in the proven sepsis group, fever in 34%, bradycardia or tachycardia in 37.7%, and need for oxygen in 30.2%. The symptoms of sepsis (decreased sucking, bradycardia or tachycardia, need for oxygen) appeared more in the proven sepsis group than in the clinical sepsis group and this was statistically significant (p<0.001). Other data relating to demographic and clinical characteristics are shown in Table 1.

**Table 1:** Demographic and clinical characteristics of patients with sepsis *p<0.05, **p<0.001, a: Chi-square, b: Kruskal Wallis

	Proven Sepsis	Clinical Sepsis	Control	P
Gender (n(%))				
Male	34 (64.2)	31 (66)	16 (40)	0.025*^,a^
Female	19 (35.8)	16 (34)	24 (60)	
Mother’s urinary infection history (n(%))	17 (32.1)	14 (29.8)	-	<0.001**^,a^
Premature membrane rupture history (n(%))	4 (7.5)	6 (12.8)	-	0.070^,a^
Delivery type				
Normal vaginal delivery	25 (47.2)	21 (44.7)	17 (42.5)	0.903^,a^
Caesarean section	28 (52.8)	26 (55.3)	23 (57.5)	
Additional disease besides sepsis (n(%))	8 (15.1)	13 (27.7)	-	0.002**^,a^
Fever (n(%))	18 (34)	20 (42.6)	-	<0.001**^,a^
Decreased sucking (n(%))	42 (79.2)	43 (91.5)	-	<0.001**^,a^
Bradycardia, tachycardia (n(%))	20 (37.7)	14 (29.8)	-	<0.001**^,a^
Need for oxygen (n(%))	16 (30.2)	10 (21.3)	-	0.001**^,a^
Number of postnatal days (Mean±Sd)	7.11±4.6	6.28±3.7	6.40±1.5	0.027*^,b^
Birth week (Mean±Sd)	38.2±1.1	38.3±1.5	39.1±0.9	0.001**^,b^
Birth weight (Mean±Sd)	3131.7±325.7	3177.9±425.7	3215.8±286.2	0.462^b^

The uric acid (3.77±1.6), CRP (17.7±10.6), white blood count (WBC) (14,562.3±5,062.7) and eosinophil (618.5±472.1) values were statistically significantly higher in the proven sepsis group than in the clinical sepsis group (p=0.022; p<0.001; p=0.002; p=0.036, respectively). The WBC, uric acid and CRP values of the patients in the control group were found statistically significantly lower than those of the proven and clinical sepsis groups. NLR was statistically significantly higher in the newborns with clinical sepsis group (3.63±14.9) than in the proven sepsis group (1.83±1.1) and the healthy control group (1.03±0.7) (p=0.002). No statistically significant difference was found between these three groups with respect to both PLR and RPR (p=0.829). The mean eosinophil value was 618.5±472.1 in the proven late sepsis group, 425.7±311.3 in the clinical sepsis group and 549.3±294.9 in the healthy control group. The eosinophil level was statistically significantly lower in the clinical sepsis group than in the control group (p: 0.036). The mean laboratory data in all three groups are shown in Table 2.

**Table 2:** Comparison of haematological parameters of sepsis and control groups *p<0.05, ** p<0.001, RDW: red cell distribution width, a: One-way ANOVA, b: Kruskal Wallis

	Proven Sepsis	Clinical Sepsis	Control	p
Haemoglobin	17.0±2.4	17.4±2.7	16.9±1.9	0.662^,a^
Red cell distribution width (RDW)	15.8±0.9	15.9±1.4	16.4±1.6	0.467^b^
White blood cell	14562.3±5062.7	12124.0±4018.8	11571.5±3899.8	0.002**^,b^
Platelets	307000±85462.8	293106.4±118351.0	318500±81936.2	0.251^b^
Red cell distribution width to platelet ratio	0.051±0.02	0.059±0.03	0.046±0.016	0.278^b^
Lymphocytes	4846.2±2128.7	5051.9±2644.8	4754.5±1510.6	0.837^b^
Platelet to lymphocyte ratio	70.3±23.6	71.5±38.9	74.0±38.1	0.829^b^
Neutrophyl to lymphocyte ratio	1.83±1.1	3.63±1.49	1.03±0.7	0.002**^,b^
Eosinophil	618.5±472.1	425.7±311.3	549.3±294.9	0.036**^b^
Mean platelet volume	9.22±0.9	8.85±1.1	8.99±0.4	0.245^b^
Uric acid	3.77±1.6	3.65±1.9	2.83±1.1	0.022**^,b^
C-reactive protein	17.7±10.6	13.3±7.5	1.44±1.5	<0.001**^,b^

It was observed that 22.6% of the microorganisms that grew in the blood cultures of newborns with proven late sepsis was Staphylococcus aureus, 28.3% Staphylococcus epidermidis, 18.9% Staphylococcus haemolyticus, 24.5% Staphylococcus hominis, 3.8% Escherichia coli and 1.9% Staphylococcus saprophiticus. A negative weak correlation was found between the birth weeks and CRP values of the newborns in all groups (r=-0.224; p=0.008). In proven sepsis, there was no significant correlation between the RPR and uric acid level or between PLR and uric acid level (p=0.993, p=0.727). A ROC curve test was conducted to find the cut-off values of RPR, PLR and uric acid for the patients in the late sepsis and control groups and their diagnostic test performances are shown in Table 3.

**Table 3:** Diagnostic test performances of RPR, PLR and uric acid in sepsis and control groups PPV: positive predictive value, NPV: negative predictive value, AUC: area under the curve, RPR: red cell distribution width to platelet ratio, PLR: platelet to lymphocyte ratio, * p<0.05, **p<0.001

	RPR	PLR	Uric acid
AUC 95%-Cl (%)	0.573 (0.486-0.656)	0.511 (0.426-0.597)	0.638 (0.552-0.717)
Cut-off	>0.05	< 56.2	>4.1
Sensitivity (%) 95%-Cl (%)	39 (29.4-49.3)	35 (25.7-45.2)	35 (25.7-45.2)
Specificity 95%-Cl (%)	77.5 (61.5-89.2)	77.5 (61.5-89.2)	95 (83.1-99.4)
PPV 95%-Cl (%)	81.2 (69.9-89)	79.5 (67.3-88)	94.6 (81.5-98.6)
NPV 95%-Cl (%)	33.7 (28.8-39)	32.3 (27.7-37.3)	36.9 (33.2-40.7)
p	0.143	0.821	0.004**

**Figure 1:**
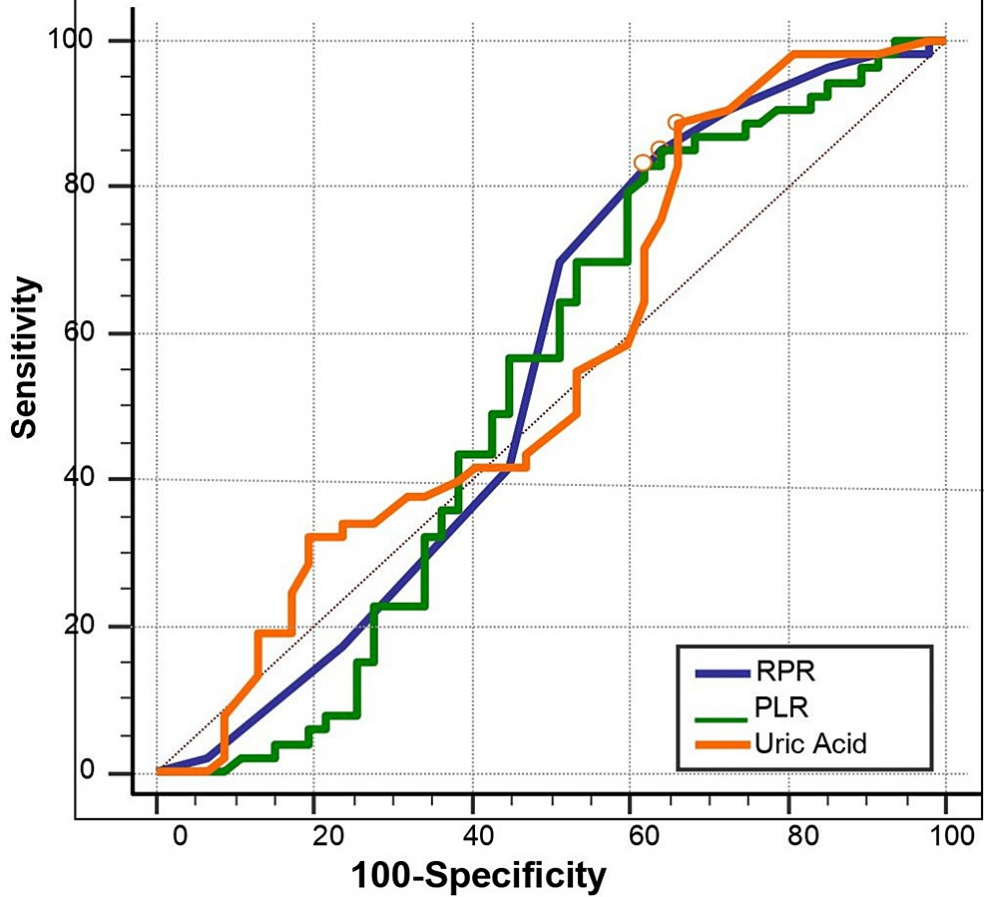
ROC curve of uric acid, RPR and PLR levels PLR: platelet to lymphocyte ratio, RPR: reticulocyte distribution width to platelet ratio

According to this, the cut-off values of the RPR, PLR and uric acid values were found to be >0.05; <56.2 and >4.1, respectively. From these cut-off values, the sensitivity of uric acid in predicting sepsis in the patients was found to be 35.0% and specificity 95.0% (p=0.004; Figure 1).

## Discussion

Approximately 400,000 newborns lose their lives due to neonatal sepsis each year and the incidences of neonatal sepsis-associated deaths have been observed to be higher in developing countries [11]. Early diagnosis and treatment are important in the prevention of morbidity and mortality in neonatal sepsis. Many non-specific clinical findings are present in neonatal sepsis and they make diagnosis more difficult and cause other newborn diseases to overlap with sepsis [12]. We aimed in our study to assess the relationship between haematological parameters and late neonatal sepsis. We found that uric acid levels had a high specificity in predicting late neonatal sepsis, whereas RPR and PLR had a low specificity in late sepsis. NLR and eosinophil levels were found higher in patients with late neonatal sepsis than in the healthy control group.

Isolation of microorganisms in blood culture is agreed to be the gold standard in the diagnosis of neonatal sepsis, but the growth of microorganisms in a blood culture requires at least 48 hours. Moreover, a blood culture turning out negative does not rule out the diagnosis of sepsis [13]. A whole blood count is a simple and readily usable test in newborns in the presence of suspected infection. In our study, MPV was higher in the sepsis group compared to the clinical sepsis and control groups, but this was not found to be statistically significant. Aydın et al. [14] found that MPV was higher in newborns with septicaemia compared to the control group and Milas et al. [15] found in their meta-analysis that MPV was statistically significantly higher in neonatal sepsis than in the control group. Unlike other studies in the literature, our study found that the increase in MPV in the sepsis group was not at a significant level compared to the control group, which may be associated with the small number of patients participating in the study and the inclusion of only those with late sepsis.

The use of NLR is known to be a fast test in detecting culture-proven neonatal sepsis. It has been reported that there is a relationship between NLR and septic shock and mortality in adult patients [16]. In the study of Omran et al. where they compared septic newborns with healthy babies, the NLR value was found higher in septic newborns [6]. Ozdemir et al. found in their study that NLR can be used to predict late neonatal sepsis in preterm babies [17]. Goldberg et al. have reported in their study that finding the NLR value together with clinical findings is useful in defining late neonatal sepsis earlier [18]. In our study, the mean NLR value in the clinical sepsis group was 3.63±1.49, which was significantly higher than that of both the proven sepsis and healthy control groups. It was thought that the patients were included in the clinical sepsis group due to the difficulty of producing the microorganism in the blood culture and therefore the negative blood culture. So, in the clinical sepsis group NLR was higher than provensepsis. A high NLR value found in our study, which involved patients with late neonatal sepsis, was consistent with the studies in the literature.

Latest studies have reported that both platelets and lymphocytes play a critical role in inflammatory processes. PLR has been reported as a systemic inflammatory marker and is known to be associated with adult cardiovascular events [19,20]. In the study of Arcagok et al., it was found to be a reliable indicator used to predict early neonatal sepsis at an earlier stage and a good alternative to the currently used parameters [4]. In newborns with proven early sepsis, PLR cut-off level was 57.7 with a sensitivity of 91.3%, specificity of 97.6%, positive predictive value (PPV) of 97.4%, and negative predictive value (NPV) of 91.8%. In the study of Can et al., the PLR cut-off value was 94.05, sensitivity 97.4%, and specificity 100%, and there was a positive correlation between NLR and PLR [21]. In our study, the PLR cut-off turned out to be 56.4, sensitivity 35%, specificity 77.5%, PPV 79.5% and NPV 32.3%, and there was no significant difference in the proven and clinical sepsis groups with respect to the PLR value in late neonatal sepsis as compared to the control group. Studies in the literature have investigated the relationship of early sepsis with PLR, but they have not investigated the relationship of late neonatal sepsis with PLR. The absence of any correlation between late sepsis and PLR is a point in our study that differs from the literature. 

RPR is an important biomarker that can be used in the diagnosis of early neonatal sepsis; it is easy to calculate and requires a very small amount of blood sample to be taken. Karabulut et al. found in their study that the level of RPR was higher in the suspected neonatal sepsis group than in the control group [12]. In our study, the sensitivity of RPR value was found to be 39%, specificity 77.5%, PPV 81.2%, and NPV 33.7%, and there was no significant difference between the neonatal sepsis group and the control group with respect to mean RPR values. There is a limited number of studies in the literature on the association of RPR level with early sepsis. As no relationship was found in our study between late neonatal sepsis and RPR, our study dealt with this subject from a different perspective. 

One of the important aspects of an acute infection is the decline in the number of eosinophils that spreads rapidly and persistently through blood circulation [22]. A decreased number of eosinophils has a high specificity in the diagnosis of early-onset sepsis. In the study of Wilar, the number of eosinophils in early-onset neonatal sepsis was significantly lower than that of healthy subjects [5]. Shabaan et al. found that eosinopenia had a sensitivity as high as 81% in predicting sepsis in adults and that eosinopenia was a very sensitive but non-specific biomarker in intensive care units [23]. In our study, the mean eosinophil count of the patients with late neonatal clinical sepsis was 425.7±311.3 and that of the control group was 549.3±294.9. The number of eosinophils was significantly lower in the patients with clinical sepsis than in the control group. The eosinopenia that occurred in the study of Wilar et al. was in the early neonatal sepsis group; there are no studies showing the relationship between late neonatal sepsis and eosinophil count. The presence of eosinopenia in the clinical late sepsis group was found consistent with the studies in the literature.

There are a lot of studies investigating the relationship between free oxygen radicals and antioxidants with neonatal sepsis [24]. However, there are a few studies so far dealing with the relationship between neonatal sepsis and uric acid which is a non-enzymatic antioxidant. In their study with 30 newborns with neonatal sepsis and 20 healthy newborns, Batra et al. found that the serum uric acid values were significantly lower in the patients with neonatal sepsis [25]. In the study of Kapoor et al. where they compared 44 newborns with neonatal sepsis and 84 healthy newborns, they found lower uric acid levels in the newborns with sepsis [26]. Aydın et al. also found that uric acid levels were lower in those with neonatal sepsis than in those without neonatal sepsis and that the uric acid levels of those with proven neonatal sepsis were significantly lower compared to the clinical sepsis group [14]. Aminiahidashti et al. have found that a high level of uric acid is a risk factor for mortality in intensive care unit patients [27]. In our study, the cut-off value of uric acid levels was found to be 4.1 with a sensitivity of 35% and specificity of 95%. The uric acid levels of the patients with neonatal sepsis were significantly higher compared to healthy newborns (p: 0.004). The uric acid levels of the newborns with proven sepsis were also higher than those of the newborns with clinical sepsis. The presence of higher uric acid levels in neonatal sepsis in our study resembles the study of Aminiahidashti et al. but differs from the other studies in the literature. Uric acid is the final product of the purine metabolism. Renal hypoperfusion is affected by excess production and tubular reabsorption [27]. Studies have reported that an acute increase in uric acid has a role in a poor prognosis in cases of critical illness [28]. Higher levels of serum uric acid in patients with late neonatal sepsis may play a supporting role in diagnosis together with other clinical and laboratory criteria. High levels of uric acid in late neonatal sepsis may suggest that clinical prognosis will be poor in these newborns. 

Our study has some limitations. The main limitation of the study is its retrospective nature. The study was made in a single centre and the sample size in the study is small. In terms of evaluating sepsis course, there was no follow-up examination of CRP and NLR. These are the other limitations of the study.

## Conclusions

NLR and eosinophil count, which are among whole blood count parameters, are higher in late neonatal sepsis compared to healthy newborns and are helpful in diagnosis. Higher levels of serum uric acid in late neonatal sepsis may provide guidance in determining prognosis by helping diagnose in an early period in patients who have other clinical findings of sepsis. We believe that with studies involving more case series, the relationship of these biomarkers with late sepsis will become clearer.

## Appendices

PATİENT DATA DIAGRAM

1. Patient number:

2. Sex:

3. Postnatal age:

4. Birth week:

5. Birth weight:

6. Delivery method:                                 a. Normal vaginal delivery               b. Caesarean section

7. Mother’s urinary infection history                      yes                                        no

8. Premature membrane rupture history               yes                                       no

9.Hypothermia/hyperthermia:                               yes                                       no

10.Tachychardia/bradycardia:                                yes                                       no

11. Increased need for oxygen                               yes                                       no

12. Decreased sucking                                            yes                                      no

13. Additional disease beside sepsis                      yes                                      no

14. Haemoglobin:……………………………………………….

15. White blood cell:……………………………………………

16. Platelets:…………………………………………………….                   

17. Eosinophil:………………………………………………….

18. Lymphocytes:………………………………………………

19. Mean platelet volume:……………………………………………

20. Red cell distribution width:…………………………………….

21. Red cell distribution width to platelet ratio:………………………

22. Platelet to lymphocyte ratio:……………………………………….

23. Neutrophyl to lymphocyte ratio:……………………………………

24. Uric acid:………………………………………………………………..

25. C-reactive protein:…………………………………………………
